# Enhancing mental health research capacity: emerging voices from the National Institute of Mental Health (NIMH) global hubs

**DOI:** 10.1186/s13033-019-0276-9

**Published:** 2019-04-03

**Authors:** Andrea Tenório Correia da Silva, Charlotte Hanlon, Ezra Susser, Graciela Rojas, Heloísa Garcia Claro, Julieta Quayle, Kassahun Habtamu, María Soledad Burrone, Maria Tavares Cavalcanti, Mona Sharma, Marguerite Schneider, Ramesh Prasad Adhikari, Tanya van de Water, Yasmin Mohammed, Anna E. Ordóñez, Soraya Seedat

**Affiliations:** 1 Departmente of Preventive Medicine, Faculdade de Medicina, University of São Paulo, São Paulo, Brazil; 2 Department of Psychiatry, School of Medicine, College of Health Sciences, Addis Ababa University, Addis Ababa, Ethiopia; 3 Columbia University, New York State Psychiatric Institute, New York City, New York USA; 4 Faculty of Medicine, University of Chile, Santiago, Chile; 5 School of Psychology, Addis Ababa University, Addis Ababa, Ethiopia; 6 Institute of Health Sciences, University of O´Higgins, Rancagua, Chile; 7 Federal University of Rio de Janeiro, Rio de Janeiro, Brazil; 8 Center for Chronic Conditions and Injuries, Public Health Foundation of India, Gurugram, India; 9 Department of Psychiatry and Mental Health, University of Cape Town, Cape Town, South Africa; 10 Transcultural Psychosocial Organization (TPO) Nepal, Kathmandu, Nepal; 11 Department of Psychiatry, Faculty of Medicine and Health Sciences, Stellenbosch University, Cape Town, South Africa; 12 Department of Behavioural Sciences, Kwame Nkrumah University of Science and Technology, Kumasi, Ghana; 13 Office of Clinical Research, National Institute of Mental Health, NIH in the United States, Bethesda, USA

**Keywords:** Capacity building, Mental health, Developing countries, Early career researchers, International collaboration

## Abstract

**Background:**

Emerging researchers in low- and middle-income countries (LMIC) face many barriers, including inadequacies in funding, international exposure and mentorship. In 2012, the National Institute of Mental Health (NIMH) funded five research hubs aimed at improving the research core for evidence-based mental health interventions, enhancing research skills in global mental health, and providing capacity building (CB) opportunities for early career investigators in LMIC. In this paper emerging researchers contextualize their experiences.

**Case presentation:**

Each of the five hubs purposively selected an emerging researcher who had experienced more than one hub-related CB opportunity and actively participated in hub-related clinical trial activities. The five ‘voices’ were invited to contribute narratives on their professional backgrounds, CB experience, challenges and successes as an emerging mental health researcher, and suggestions for future CB activities. These narratives are presented as case studies. CB activities provided broader learning opportunities for emerging researchers. Benefits included the receipt of research funding, hands-on training and mentorship, as well as exposure to networks and collaborative opportunities on a global scale. To overcome ongoing challenges of access to funding, mentoring, networking and global exposure, the emerging voices recommend making mentorship and training opportunities available to a wider range of emerging mental health researchers.

**Conclusions:**

Investing in CB is not enough to ensure sustainability and leave a legacy unless it is accompanied by ongoing mentorship and international exposure. Financial investment in building research capacity, promotion of mentorship and supervision, and international networking are essential to yield well-prepared young investigators in LMIC as experienced by these rising stars. Governments and policymakers should prioritize educational policies to support the continuous development and international engagement of emerging researchers. This can advance strategies to deal with one of most important and costly problems faced by healthcare systems in LMIC: the mental health treatment gap.

## Background

Over the last 20 years, the global burden associated with non-communicable chronic diseases (NCD) rose from 47 to 54% [1]. This rising burden of NCDs is projected to impact negatively on economic growth as a consequence of decreasing productivity due to disability and out-of-pocket expenditures by families and by overloading health and welfare systems [2, 3]. Mental disorders account for an increasing proportion of the burden attributable to NCD, causing a high degree of individual and social suffering [4]. According to the Global Burden of Disease study, mental disorders were responsible for 157.9 million disability-adjusted life years (DALY) lost in 2005, and 173.2 million in 2013, representing an 9.7% increase in 8 years [5]. The negative impact of mental disorders on healthcare systems is particularly important in low- and middle-income countries (LMIC) [6]. Resources provided in LMIC to tackle the burden of mental disorders are insufficient, inequitably distributed, and inefficiently used [7–11]. As a consequence, many LMIC have a treatment gap (i.e., the proportion of individuals with mental disorders who do not receive health care) larger than 75% [11]. Even when available, treatment and care often is neither evidence-based nor of high quality [11].

There are many ongoing efforts to mitigate this gap and scale up healthcare services for people with mental disorders [12, 13]. One example is the World Health Organization’s (WHO) Global Mental Health Action Plan, which focuses on improving access to mental healthcare through adoption and implementation of policies and plans that promote the integration of mental health into general health care [14]. Linked to this, WHO launched the Mental Health Gap Action Programme (mhGAP) [15–17] which seeks to reduce the burden of mental disorders and to enhance the capacity to scale up services for people with mental disorders in LMIC [15].

Successful scale up of mental healthcare is the joint responsibility of governments, health professionals, civil society, communities, and families, with support from the international community. To support these efforts, there is a need to build the capacity of human resources to support health system strengthening, as well as in the direct provision of mental health care [2, 11, 18]. Fricchione et al. have highlighted that “the approach to building a mental health infrastructure for LMIC must be bidirectional. In addition to nurturing career trajectories in academic medical centers residing in high income countries (HICs), efforts need to be made to develop a cadre of home-grown, in-country professionals and basic mental health workers. Linking these efforts is critical” [19].

Initiatives that help develop or provide access to resources and the benefits of research collaboration to these countries are important tools to overcome many of the existing barriers [20]. Research capacity-building (CB) activities in particular could make an important contribution to the transformation of mental health services in LMIC by creating opportunities to train young professionals to become independent and competitive on the global scientific stage, generate collaborative international networks, produce partnerships between research teams, local implementers and policymakers, conduct contextually relevant research with a higher chance of country ownership and translation into practice, and provide mentoring to budding researchers [21–23]. Despite this, research capacity-building experiences based on LMIC and HIC partnerships have been scarcely investigated and reported. Moreover, the perspectives of emerging researchers who have participated in CB activities, regarding barriers and facilitators, have rarely been addressed. Examining this issue may yield valuable information to inform future CB partnerships, and their impact on mental health care, particularly in LMIC.

The NIMH funded five collaborative hubs for international research in mental health in LMICs (https://www.nimh.nih.gov/about/organization/gmh/globalhubs/index.shtml). The Hubs were awarded through a competitive NIHM grant call and represent partnerships between researchers in HICs and LMICs. The aim of this initiative was to address the mental health research gap [24], increase research output and enhance access to CB opportunities in these settings [23, 25].

- Africa Focus on Intervention Research for Mental Health (AFFIRM) includes researchers from Ethiopia, Ghana, Malawi, South Africa, Uganda, and Zimbabwe (http://www.affirm.uct.ac.za).
- Latin America Treatment and Innovation Network in Mental Health (LATIN-MH) includes Brazil, Colombia, Ecuador, Guatemala, Peru, United Kingdom and the United States (http://www.latinmh.com.br/).
- Partnership for Mental Health Development in Sub-Saharan Africa (PAM-D) has forged collaborations between Ghana, Kenya, Liberia, Nigeria, and South Africa.
- Regional Network for Mental Health Research in Latin America (RedeAmericas) comprises Argentina, Brazil, Chile, Colombia, and USA (http://cugmhp.org/research/redeamericas/).
- South Asian Hub for Advocacy, Research and Education on Mental Health (SHARE) consists of representatives from Afghanistan, Bangladesh, India, Nepal, Pakistan, and Sri Lanka (http://www.centreforglobalmentalhealth.org/projects-research/share-south-asian-hub-advocacy-research-and-education-mental-health).

While the monitoring and evaluation of the CB initiatives within the hubs have been published [23, 25], the report did not include the perspectives of researchers who participated in these activities. This manuscript therefore, provides emerging researchers with an opportunity to describe their experiences of research CB opportunities.

## Case presentation

Each of the five hubs purposively selected one emerging researcher, considered to be an emerging leader in global mental health, who had (i) experienced more than one hub-related CB opportunity and (ii) participated in hub-related research activities. The five voices were invited to furnish narratives describing their professional backgrounds, experience of CB activities, challenges and successes as emerging health researchers, and suggestions for future CB activities and were provided with a set of question prompts to frame their narratives (What is your background? What capacity building activities have you participated in? What Hub-related activities are you involved in? How do you feel about being identified as an emerging researcher? What was your experience of CB activities? What do you perceive as barriers for other emerging researchers in your setting? What would be your recommended next steps? What are the key learnings from your experience as a researcher in an LMIC?). Emerging researchers from the five Hubs ranged in age from 29 to 37 years (3 females, 2 males), had a minimum of a Master’s degree, and worked under the guidance of the Capacity Building coordinator of their respective Hub. We describe five case studies here in the form of first person narratives that are presented as excerpts. The five ‘emerging voices’ are described in Table 1.

**Table 1:** Emerging voices

Narrator, age, gender	Hub (duration) and country	Professional background
Kassahun Habtamu, 37, male	AFFIRM (4 years), Ethiopia	Ph.D. (Mental Health Epidemiology), Post-doctoral fellow, AMARI
Heloísa Garcia Claro, 29, female	LATIN MH (2 years), Brazil	Postdoctoral Fellow (Nursing)
Yasmin Mohammed, 32, female	PAM-D (3 years), Ghana	M.Phil. (Clinical Psychologist), Ph.D. Candidate
María Soledad Burrone, 37, female	RedeAmericas (2 years), Argentina	MD (community physician), MPH, Ph.D.
Ramesh Prasad Adhikari, 33, male	SHARE (2 years), Nepal	Masters (Population Studies and Sociology/Anthropology)

## Learning from CB activities

### AFFIRM

As part of AFFIRM, I have been involved in several capacity building (CB) activities. Most importantly, the AFFIRM CB program supported me by covering all my research expenses for my Ph.D. I attended two AFFIRM annual meetings (one in Ethiopia and one in Malawi), where I presented my Ph.D. work and received feedback from world class scientists. The AFFIRM CB program funded my attendance at two AFFIRM short training courses—one on operational research and one on randomized controlled trials in mental health. As an AFFIRM Ph.D. student I was funded to attend the International Mental Health Research Conference organized by the University of Malawi, College of Health Sciences in 2015. The AFFIRM network provided access to a world class professor as an external supervisor for my Ph.D. The expertise, advice, and feedback that I received from Prof Martin Prince, particularly on scale development, validation and psychometric analysis was really unique and offered a tremendous opportunity. The CB activities that I was involved in through the AFFIRM project commenced at the beginning of my Ph.D. (November 2012) and are continuing. This includes training field workers in data collection, project coordination, supervision and monitoring of fieldwork activities, data management (data entry, data cleaning and data analysis), literature searches and review and publication of peer-reviewed papers. Over the 4-year period within AFFIRM, I have published four peer-reviewed papers (three as leading author and one as a second author). Being part of AFFIRM has helped me a lot to get these papers published through mentorship, supervision and short courses. Although I was not part of a regular mentoring program, my Ph.D. supervision paralleled a mentoring process, with face-to-face support and regular on-site meetings. The spirit of discussion during these mentoring and supervision meetings was about professional growth and not just focused on the Ph.D. pursuit. If I had not received this input during my Ph.D., I would not have published a single paper.

### LATIN-MH

I started in Latin MH in 2015, just after finishing my Ph.D.: first as Field Work Coordinator and, soon after, as Data Center Coordinator. I helped to build our database and digital data collection forms, establish quality assurance methods and ensure the safety of data, analysis, as well as data validation rules, and tried to make the system user-friendly. I was given the opportunity, as field coordinator, to plan a big field research project with close supervision. I learned a lot about protocol development, human resources management, documents and regulations for trials, among other issues. As a data center coordinator, I also had to learn to contract and negotiate with programmers, manage their work and monitor their activities.

The Latin-MH group discussions that I have participated in help young researchers to access scientific information and interact with senior professionals. This is what I look for as a researcher—to work as a bridge between the university and clinicians working in health services.

I was invited to do a presentation on human subject research ethics to our Latin-MH staff to help them prepare for the trial and get the necessary certifications. In 2016 I also worked as a temporary teacher at the University of São Paulo, teaching undergraduate students and residents about mental health. I had the opportunity to meet with some of the best researchers in mental health during the Collaborative Hubs Meeting held in São Paulo in September 2016, an experience like no other.

I feel honored and think it is a great responsibility to be included here as an emerging researcher. The Capacity Building component of the Hub favors professional growth beyond research activities alone. Writing is one of them and I was able to work with the PIs (Paulo Rossi Menezes and Ricardo Araya) on two manuscripts on the development of the study protocol, and realized how important it is to benefit from the experience of senior researchers.

### PAM-D

Over the past 2 years, I have had the opportunity of participating in three capacity building workshops organized by the Partnership for Mental Health Development in Sub-Saharan Africa (PaM-D) Hub. My very first capacity building opportunity on PaM-D was the scientific writing workshop “Getting your paper published in peer reviewed international journals”, held in Johannesburg, South Africa from March 27–30, 2015, during which I learnt about phrasing a research question, identifying data analytic methods to match my hypothesis, putting together a good abstract, knowledgeably preparing for a poster/oral presentation as well as identifying peer-reviewed journals most suitable for my research on “Internalized Stigma of Mental Illness in Ghana”.

It was a totally different experience from all the workshops that I had participated in until then because it required participants to be actively involved in the knowledge impartation process. Every new topic that was introduced was followed by either an individual or group activity that required you to put what you had just learnt into practice—right then and there—so that the facilitators could ascertain whether or not learning had indeed taken place. Personally, I would say this hands-on approach was the reason that all our objectives were achieved by the end of the workshop. The practical nature of the sessions ensured that we made good use of the time allocated for every topic we had to cover. Further, working with deadlines ensured that we applied what we had been taught immediately after to ensure that learning had taken place. The feedback we received during the mock editorial boards and subsequently from our facilitators, Prof. Soraya Seedat and Prof. Francis Creed also gave us more insight into what it took for a paper to be either accepted or rejected by international journals.

In July 2016, I had another opportunity to attend one more Capacity Building Qualitative Research workshop organized by the PaM-D Hub, at Stellenbosch University in Cape Town. This workshop was organized based on the expressed need of early career researchers who attended the first workshop on training in qualitative research methods, since most of us had very little or no experience at all with qualitative research. The qualitative workshop also followed the practical format of the initial workshop, with the knowledge gained on designing questionnaires and conducting one-on-one interviews and focus groups being applied and demonstrated among attendees during the classroom sessions. By the end of the workshop, the prospect of embarking on qualitative research and mixed methods seemed less daunting because we knew we could always have access to ongoing support from our facilitators (Dr. Donald Skinner and Prof. Soraya Seedat).

The third workshop—the 2nd scientific writing workshop—was a follow-up to the very first scientific writing workshop in March 2015. It was designed to respond to the recommendation of attendees of the first workshop to allocate more time and one-on-one sessions with facilitators. The overall aim was to identify research designs and data analysis methods that were in line with the various hypotheses. To this end, two additional facilitators (Dr. David Macharia and Prof. Martin Kidd) were invited to assist attendees with their research designs and data analysis, respectively. By close of the last day, the feedback from all attendees was just as good as the feedback received after the first scientific workshop because of the practical nature of the sessions and the efficient use of time. We therefore recommended that a follow up workshop be organized in which more time could be allocated to data analysis, with other young researchers from Sub-Saharan Africa invited to participate.

Apart from the knowledge gained during the various workshops, I have also received a lot of training on instrument administration, documentation and regulations for randomized controlled trials, commensurate with my duties of coordinating, managing and monitoring field activities on the PaM-D project, and in accordance with the research protocol and ethical guidelines involving human participants.

### RedeAmericas

In 2011, I obtained my MPH from the University of Córdoba, Argentina and I started my Ph.D. I also received a 2-year Career Development Award from RedeAmericas (RA, NIMH U19 grant, Dr. Susser PI). This was a very exciting experience for me. During the first year, I attended a course on epidemiology by the International Epidemiology Association in Lima, Perú, along with other RA awardees from Chile to Colombia. We quickly established a collaborative network which grew over time to include new RA Awardees and other early career investigators from Latin America.

Through the RA Career Development Award fellowship, I received mentorship and guidance from Drs. Ruth Fernandez and Ruben Alvarado to map out my career path in mental health research. I also received support to overcome the limitations and barriers for emerging researchers which are common in my setting. This included improving my English and writing skills (as we do not receive this training at medical school), and receiving economic support to attend conferences on mental health. At the same time, I received first-hand training from senior RA investigators (Drs. Conover, Valencia and Susser) on the Critical Time Intervention-Task Shifting (CTI-TS), a community-based intervention to help people with psychosis to actively engage in treatment and to integrate into their communities. Then, I had the incredible opportunity of applying this knowledge by collaborating on the implementation of the CTI-TS in Córdoba.

Being involved in RA was a great experience for networking. I participated in many conferences on Public Health, Epidemiology and Mental Health in Latin-America, the US and Europe, and was involved in the organization of a few conferences in Latin America. This gave me the opportunity to interact with many well-known researchers in mental health. I also attended all annual RA meetings along with other RA awardees and investigators, and have made several extended visits to the Department of Epidemiology at Columbia University.

### SHARE

I participated in the SHARE course on Mental Health Services Research in a Humanitarian Context, which is a 2-year part time course conducted by SHARE hub in collaboration with University of Liverpool and Johns Hopkins School of Public Health.

The course is an innovative and skills based program that has been designed to build the research skills of early career researchers who are already working in humanitarian settings. It is based on the Design, Implementation, Monitoring and Evaluation (DIME) Model, and is divided in four different modules with clear linkages to the practical aspects of conducting research and implementation. The course consisted of theory classes (conducted at course site in Pakistan) followed by practical work that is carried out by the trainee in their original work context. The course offered both financial and academic support to trainees to conduct research. Implementation of learnt skills was further supported by regular supervision and technical support provided by the mentors and tutors. An important aspect of the course was the online learning forum that provided a platform to trainees to share and clarify issues related to research design and implementation, by creation of a peer network and facilitation of a virtual dialogue with course faculty and mentors.

The 2-year course designed by SHARE has significantly helped me in developing my skills, interest and career as a public health researcher in humanitarian settings. After participating in the course, I learnt other key skills, such as conducting qualitative assessments to determine the priority issues for further research, and the selection and adoption of interventions based on their feasibility, acceptability and significance for the community. The course provided extensive training in the development and validation of new tools based on initial qualitative work. Further, I learnt to evaluate the appropriateness (feasibility, acceptability and impact) of a specific intervention in target communities.

Participation in the course has had a positive impact, both on my academic and professional life. Since participating in the course, I have published 7 papers in international peer reviewed journals as first author and contributed as a co-author to 4 papers that have also been published in international journals. One of the published papers was developed based on the first phase of the SHARE study, undertaken as part of the humanitarian course. My course mentor supported publication of my research work, by closely supervising and linking me with senior researchers and funding sources to conduct additional research in the same area. My career has further benefitted from participation in the course as I was promoted to lead research projects at my organization.

The course has positively impacted my career track as a public health professional, as I am applying for admission to a doctoral program this year. The intervention developed during the SHARE course was effective in the sample population in which it was tested. I am now working on applying for grants to scale up the intervention in a large population.

## Challenges and recommendations

In this section, we present selected excerpts of the narratives of emerging researchers that relate to the challenges they faced in their local research environments. Furthermore, we identify expectations and future recommendations from these narratives.

### AFFIRM

Emerging researchers in the Ethiopian setting face a number of challenges and barriers. The single most important barrier is inadequate research funding. Other barriers include accessing a Ph.D. supervisor, absence of hands-on and focused short training courses, lack of access to international exposure and limited skill and commitment to publish papers. My involvement in AFFIRM research and CB activities helped me to overcome these challenges and barriers within a nurturing and facilitating context. I am now well on my path as an academic and researcher in mental health, as evidenced by my selection as a Wellcome Trust-funded AMARI postdoctoral fellow (http://www.amariconsortium.org). Crucial factors that contributed to my success were self-learning, excellent local and international supervision and mentoring, good research funding, and international exposure through collaboration and exchange visits. In reflecting on my journey within AFFIRM, key factors to consider for other emerging researchers in LMIC contexts include access to needs-driven and hands-on short courses, enhancing the capacity of local supervisors and mentors, providing networking and collaborative opportunities and facilitating funding and exchange visits.

### LATIN-MH

One barrier for emerging researchers is finding time to work on papers and presentations. It takes a lot from an emerging researcher to get a randomized controlled trial off the ground and there is little time to improve your CV. I plan to become a faculty member at a good university in the near future. Right now, I want to benefit more from the experiences of LATIN-MH senior researchers by participating in new projects and the writing of funding proposals. It has been a fascinating opportunity to work within a setting of international high standard research. I greatly recommend that young researchers pursue this kind of opportunity. It is amazing how it can expand one’s horizons.

### PAM-D

Over the last 14 months, I have submitted two articles to international journals which are currently in press, won two travel fellowship grants for early career researchers to attend the 2016 and 2017 World Psychiatric Association International Congress in Cape Town (South Africa) and Berlin (Germany), respectively. I have also put together a proposal for my Ph.D. studies based on the knowledge acquired from my participation in capacity building activities. It is therefore my hope that other young researchers will also be afforded such opportunities to learn and evolve, just as I have had the opportunity to keep learning and evolving both as a clinician and a researcher.

### RedeAmericas

Over the years, the support and training that I received from RA has crystallized into many important achievements. I completed my dissertation project, which was the first study of the prevalence of mental disorders in Córdoba, and completed courses for my second Master's degree, focused on mental health. With a nomination from Dr. Susser, I won the prestigious Lisa Oehler Award from Goettingen, Germany, to support the further development of my research career. Finally, I was appointed as Professor and Young Researcher at the Institute of Health Sciences, University of O´Higgins, in Chile, and was selected to co-coordinate the CB activities in RedeAmericas 2. Being involved in a collaborative network and participating in research activities alongside RA investigators has been extremely important for my development. I would encourage those interested in developing a career on mental health research to engage in global collaborations and explore opportunities for active research involvement to develop the skills that they need.

### SHARE

I am grateful to the SHARE consortium for providing me with a fellowship and a follow up grant after successful implementation of first, second and third phases of SHARE fellowship (1st phase: qualitative assessment; 2nd phase: intervention development and program planning; 3rd phase: instrument development, validation and use in baseline studies).

Overall, I am happy with the content and process of the training course. I would like to suggest that instead of conducting training in one country only, the different phases of the training be done in different country locations. This would have helped to hone cross-learning and contextual observation skills.

## Discussion and conclusions

The five ‘voices’ highlight a number of overlapping key challenges they face as emerging researchers. Each complete narrative was reviewed by the CB coordinator of that Hub. Together with the CB co-ordinator, emerging researchers identified themes, checked points of agreement and disagreement, reviewed aspects of their CB experience that were challenging, and highlighted recommendations for future CB activities. Table 2 shows a summary of the challenges along with recommendations.

**Table 2:** Key challenges facing emerging researchers and recommendations

Challenges	Recommendations
Access to funding	Assisting established researchers to write their grant applications and gaining experience in the process (this could be linked to a mentoring program for early career researchers) Fostering collaborations between established researchers in high income countries and early career psychiatrists in LMIC to enhance access to funding opportunities
Access to mentorship	Fostering collaborations with established international researchers using technology to make mentorship more accessible Providing continuing mentorship after completion of CB activities and enhancing learning process and researchers motivation Funding international exchange visits of early career researchers across high and LMIC Promoting international networking opportunities
Lack of research specific skills	Providing training in project-specific and context-relevant skills, including training on online platforms and short courses Supervising training in the practical applications of CB activities (e.g. a course of qualitative research methods could be twinned with a hands-on course on qualitative data analysis)
Scarcity of hands for supervised research training	Providing supervised field training on research planning and implementation

**Fig. 1:**
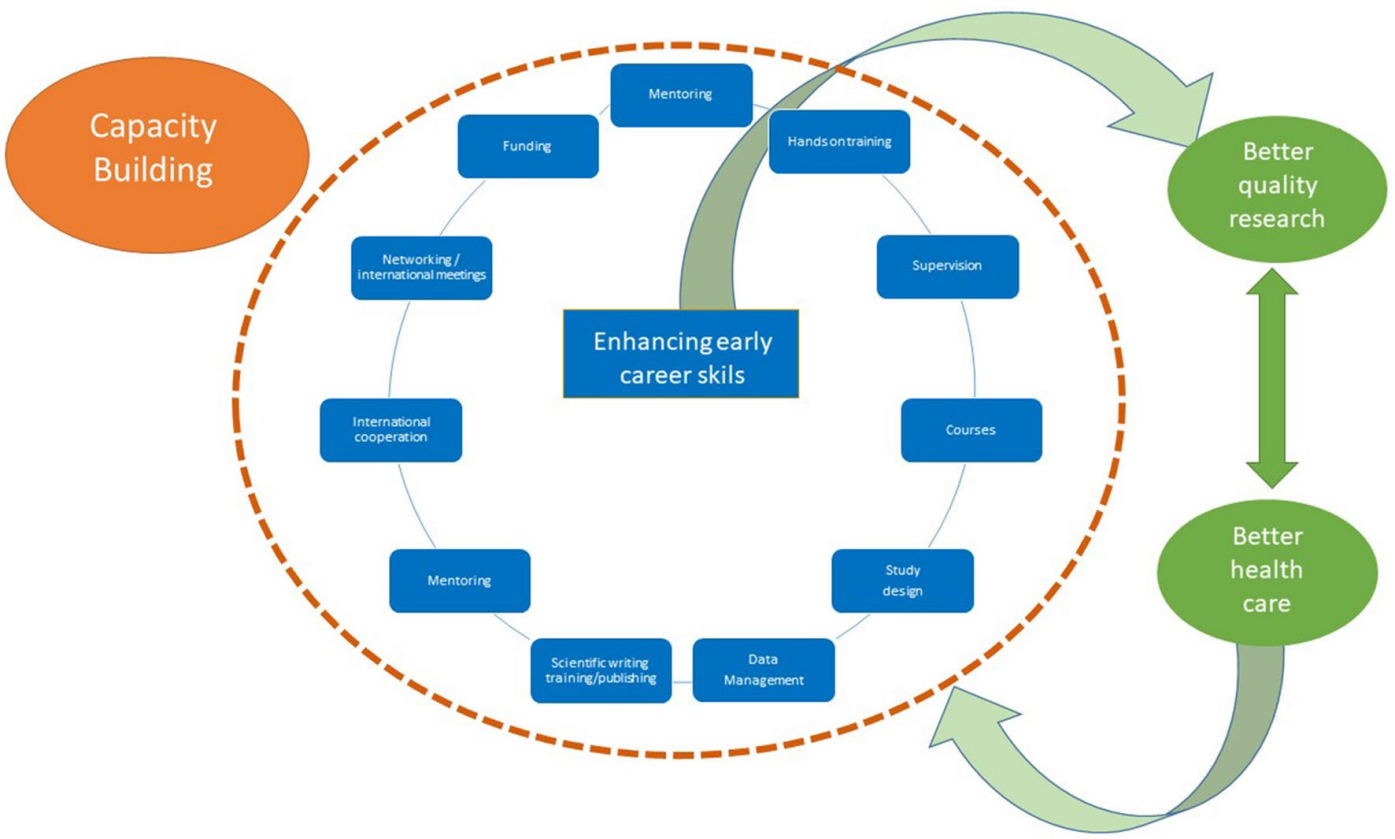
Capacity Building strategies to enhance early career skills reported by the five emerging voices from the NIMH Hubs

Capacity building requires a pragmatic, scientific, and ethical approach. These young researchers have to build and perfect their skills and have the opportunity to network and collaborate with other hubs. While doing so, they can also meaningfully increase research capacity in their region and contribute to the development of better quality mental health services for the population. Of no less importance is the fact that these young researchers will, in a short time, also contribute to increasing research teaching capacity. This strategy of boosting local, relevant research in currently underrepresented regions and populations is not only helpful in addressing the treatment gap in mental health in LMICs, but also in increasing scientific knowledge globally. Figure 1 proposes a schematic representation of this.

This article presents individual-level perspectives of five early career investigators in global mental health. Whilst their views may not be representative of all other early career researchers who engaged in CB activities in the Global Hubs, these case studies can be informative for understanding the function and impact of CB partnerships and activities at an individual-researcher level. Another limitation is that these rising stars were asked to provide these narratives by their mentors which may have contributed to social desirability bias. To overcome this, they were encouraged to be frank about both the benefits and challenges they experienced.

Overall, early career researchers in LMIC have to juggle multiple roles and responsibilities. A rational approach to capacity-strengthening that marries the focus and format of research training and supervision activities with local needs and priorities is key to its success. More dedicated investment in training early career mental health researchers is central to the sustainable development and promotion of both the mental health research agenda in LMIC and globally. The experiences of these emerging researchers working in diverse cultural settings exemplify the importance of “learning by doing” and the importance of accessing scientific know-how and research resources. A number of themes cross-cut the gain of new knowledge and diverse skills: the focus on research methods training, the perceived benefits of strengthening research skills whilst immersed in a collaborative research project, the importance of collaboration, supervision, mentorship and international exposure, and the impact on individual level research output and career development.

Finally, educational and funding policies to provide continuous support for the development of early investigators, including international cooperation, should be prioritized by governments and policymakers. This is particularly important in LMIC where the consequences of poor mental health may be even greater than in high-income countries, because of the lack of social protection safety nets, access to care, and the relationship between poor mental health and poverty. Furthermore, strengthening the investment in capacity building research on mental health may contribute to building strategies to deal with one of the most important and costly problems faced by healthcare systems globally but more especially in LMIC: the mental health treatment gap.

## Abbreviations

AFFIRM: Africa Focus on Intervention Research for Mental Health
CB: capacity building
DALY: disability-adjusted life years
HICs: high income countries
LATIN-MH: Latin America Treatment and Innovation Network in Mental Health
LMIC: low and middle-income countries
mhGAP: Mental Health Gap Action Programme
NCD: non-communicable chronic diseases
NIMH: National Institute of Mental Health
PAM-D: Partnership for Mental Health Development in Sub-Saharan Africa
RedeAmericas: Regional Network for Mental Health Research in Latin America
SHARE: South Asian Hub for Advocacy, Research and Education on Mental Health
WHO: World Health Organization’s
